# Telling it like it is

**DOI:** 10.7554/eLife.04902

**Published:** 2014-10-17

**Authors:** Alison Woollard

**Affiliations:** Department of Biochemistry, University of Oxford, Oxford, United Kingdomalison.woollard@bioch.ox.ac.uk

**Keywords:** outreach, public engagement, social media

## Abstract

Following a year of public engagement activities associated with the Royal Institution Christmas Lectures, Alison Woollard explains why scientists need to communicate with the public.

I have just come to the end of a year of activities related to delivering the 2013 Christmas Lectures at the Royal Institution in London. My lectures were the 185th in a series (but only the fifth presented by a woman) that was established by Michael Faraday in 1825 to present a scientific topic to the general public, especially young people, in an entertaining way (Figure 1). The lectures have been televised over the Christmas period in the United Kingdom since 1966. My topic was ‘Life Fantastic’ and my lectures covered developmental biology, genetics and evolution.

**Figure 1.**
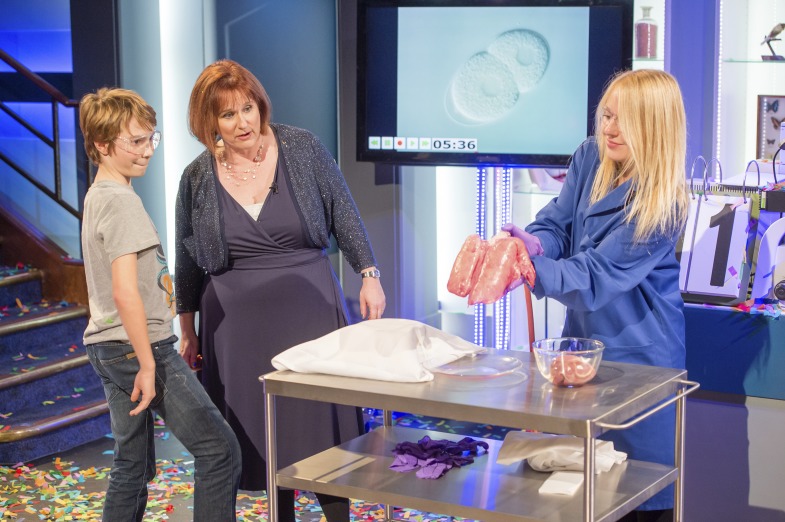
The author (centre) in full flow on the set of ‘Life Fantastic’, discussing the human lung. Scientists involved in public engagement have the power to inspire future generations of scientists, influence government policy on controversial topics, and share their passion for science with a wide range of people.

FIGURE CREDIT: Copyright Paul Wilkinson (http://www.paulwilkinsonphotography.co.uk).

Over the year, I have climbed an extremely steep learning curve in public engagement, which has taken me from the Green Man Festival in the mountains of Wales to the Mediacorp TV studios in Singapore. I've been involved in many types of public engagement: from the razzmatazz of the Lectures themselves with all the surrounding media coverage, through to experimenting with various forms of social media and online activities—and more. This year has been a rollercoaster of excitement, fear, fun and incredulity, in fairly equal measures. So perhaps it's time to reflect on the general areas of science communication and public engagement: firstly, was it worth it?

The impression that has stayed with me the most is people's hunger for knowledge, and their excitement when they make a new connection. Not just in relation to scientific ideas, but about the scientific process. I remember explaining to the film crew who recorded the Christmas Lectures how you could take a gene out of any organism, put it into another (worms, in this case) and find the gene still works. They were utterly spellbound: not just by the overall result, but by the details—how ‘getting the gene out’ and ‘putting the gene in’ is actually done in the lab. Then there was the 10 year old who wanted to know whether the chicken or egg came first and wasn't completely satisfied with my explanation, the 87 year old who wrote to ask me how cells know where they are ‘within the amorphous blob that everything starts from’, and the 20-something festival-goer who stopped me after a talk and wanted to know what regulates transcription factors. Music to the ear of a developmental biologist!

If the thrill of developing someone's sense of wonder about the world hasn't convinced you that public engagement is worth the effort, think about the ‘controversy’ surrounding climate change. A study in 2010 showed that over 97% of active climate researchers agree that climate change is occurring and that it is primarily induced by human activity (Anderegg et al., 2010). Unfortunately, however, there appears to be a disturbing chasm between this consensus and the general public's perception of the research community, which has largely focused on the incorrect notion that there is rampant scientific disagreement about the facts (Somerville and Hassol, 2011). The media don't always help either—often giving opposing sides of the debate equal airtime in the interests of ‘fairness’ (but certainly not worrying about matching the quality of the arguments on each side).

There may well be complex reasons for the public's confusion, not least economic uncertainty and a well-orchestrated campaign of disinformation by particular public figures and lobby groups. Climate scientists must therefore communicate a clear message, and they need to repeat this every time a fallacious claim is used to discredit the science. This can be a relentless and exhausting task, but it must be done. A breakdown in direct communication of science to the public can lead to widespread mistrust and a misunderstanding of scientists and their research. In the vacuum created by such a communication failure, opinion and rhetoric based on cherry-picked data can hold sway over weight of evidence and logical argument.

Compare the coverage of climate change with that relating to so-called ‘three parent’ babies. Are these a medical breakthrough or a slippery slope to designer babies? Here is an example where the details of the biology are so important, and must be communicated thoroughly and with crystal clarity.

Around 1 in 2000 people are estimated to have inherited mitochondrial diseases, which are currently incurable. These affect the mitochondria in a cell, which produce energy—in a way, they are the batteries of a cell. To eliminate these diseases, it is suggested that the faulty mitochondria in the egg cell from the mother are replaced by healthy mitochondria from another woman's egg cell. However, mitochondria have their own genome, entirely separate from the DNA in the nucleus. That is a surprise to most people (even some biologists!). Therefore, it is said that the resulting child has three parents as it receives genes from three people—mother, father, and the woman who donated her mitochondria.

To contextualize this science, it is important to know that mitochondrial DNA makes up less than 0.2% of our total DNA, and only encodes our cellular energy generating machinery. This means there is no chance the resulting baby will inherit the donor's personal characteristics—like eye or hair colour. So this technology is not about creating designer babies at all, it is about building on our expertise in in vitro fertilisation to eliminate devastating diseases.

This issue, unlike climate change, seems to be fairly well understood by the public, with informative and accurate press coverage, on the whole, that steers away from sensationalist ‘Frankenstein baby’ headlines. This is despite the fact that the ‘new’ mitochondria will be inherited by all subsequent generations. Mitochondrial replacement is therefore a kind of germ line gene therapy—a traditional line in the sand for some who hold that it is unethical to modify the genomes of those who are not in a position to consent.

Last year in the United Kingdom, the Human Fertilisation and Embryology Authority (HFEA) undertook extensive public consultation on mitochondrial replacement, finding widespread support for it (Human Fertilisation and Embryology Authority, 2013). High quality public engagement has played a large part in generating this positive, settled public view—accurate, detailed information painstakingly described in a succession of careful and reflective interviews and articles by the scientists involved. The proposed regulations that will allow this procedure to be carried out, under license from the HFEA, have been published and are due to be debated by the UK parliament later this year.

So influencing public policy seems like a prime reason for spending time on public engagement activities. There are others. Much of our research is publically funded, so surely the public has a right to know what all the money is being spent on. If the public understands and sees the worth in what we do, then this will ultimately filter through to government policy, and the very funding on which we all rely.

Science must be more strongly embedded in society, not ‘just’ as a crucial driver of economic competitiveness and improved healthcare, but also as an important cultural ambition; the pursuit of knowledge is a high calling and a defining human activity. We strive to understand the world around us, and in doing so derive a sense of satisfaction, wonder and joy that the whole of society can share in.

We also need to inspire the next generation at a time when the stakes are very high for our young people. They come out of university into an uncertain jobs market with massive debts. Why should they study particle physics, or bird migration? Your passion is the answer to that question, and you must let that shine through.

If this has fired your enthusiasm for public engagement, there are plenty of ways you can get involved. There are also a large number of resources online with tips and advice for how to communicate effectively (Box 1).

Box 1. **How to tell it like it is**

Public engagement is a big growth area and there are a huge number of different ways you can get involved. They are all different, so play to your own strengths.

Advice on how to communicate well with a range of audiences can be found in a number of places. A good place to start is Communicating Science, a document produced by the European Commission. The British Science Association, Science Media Centre and National Co-ordinating Centre for Public Engagement websites also contain a variety of tips, training sessions, and information about UK-based events to get involved in.

The internet provides an easy way to communicate your science to (potentially) the whole world without even having to leave your lab. Simple ways of getting started include starting your own blog, or using Twitter. There are also online engagement platforms like http://imascientist.org.uk, which runs several events a year where scientists at all stages of their career answer questions posed by school children.

If you'd rather communicate with your audience face-to-face, many universities and research institutions have schemes that can set up talks in schools or public lectures. There are also locally-based organizations in many areas that organize science-based open mic nights, which allow you to communicate science in a wide range of formats—even interpretive dance! In the UK, Science Show Off holds open mic nights in several cities, as does Bright Club, the ‘thinking persons variety night’.

The UK has one of the most extensive science communication communities, but there are also opportunities to get involved in the rest of the world. There are an increasing number of national organizations with advice and participation opportunities: for example Inspiring Australia. ‘Pint of Science’ festivals, in which cutting edge research is discussed in the pub, are held in the UK, US, France, Switzerland, Ireland and Australia. Finally, if you're feeling competitive, Fame Lab is an international competition where entrants have three minutes to talk about a scientific topic. Heats are currently held in over 20 countries (eligibility criteria vary between countries).

My advice? Find out the questions before you prescribe the answers! Importantly, remember that engagement is a two-way process. It is about being interested in people and listening to what they say. Science is not an elitist club that most people cannot join—and public engagement should not be an overt ‘knowledge dissemination strategy’. Good science communication is a huge learning experience for the communicator as well as the audience—it certainly was for me.

Finding a different relationship with your science can be an extremely creative process. You have to get inside the heads of your audience and go with them on their journey, seeing the problem from all perspectives. And you can only do that if you have thought very deeply about your science and worried a lot about the bits that don't quite fit. Don't be afraid about exposing these—your audience will find them fascinating. They need to know that you are all on a shared journey together, no matter how old they are or why they are listening to you. If they think this, they will connect with you and you will connect with them. They will have the confidence to know that understanding the answers is within their reach. It all comes down to good story telling. That is the beginning, the middle and the end of good science communication.

Some scientific concepts are pretty tricky so you need to learn how to break them down. Keep the story tight but don't shirk on the crucial details. When preparing for my Christmas Lectures I made the decision very early on that steering away from molecular mechanisms because they are too complicated would be a mistake, so knew I would have to bring in the idea of regulated gene expression. That is a concept with a lot of associated baggage—DNA, genes and transcription factors, to name but a few. The story needed to be built up incrementally, and with a completely logical narrative arc, otherwise the audience would be lost. That was the greatest challenge of the series for me (well, that and Mendelian Genetics!). You have to be very self-critical. You must listen hard to your own explanations—to your own narrative—to see where the holes are, and where it is hard to follow. Simple analogies can be extremely helpful, as can interactive games and other kinds of audience participation. And wow facts—did you know the human brain contains 86 billion neurons (Azevedo et al., 2009)?

Of course, it's easy to say that we're all too busy to get involved in public engagement, but this is important work. Particularly in the biological sciences, I can really feel the vital importance of effective public engagement, especially at a time when the UK government demands ‘impact’ and some have issues with the potential implications of research in genetics and molecular biology. But above all, I really can't overstate the sheer enjoyment of sharing my sense of wonder and excitement about the biological world. As a viewer wrote: ‘Hubby and I have been glued to the screen and amazed that we have now even the smallest grasp on such a complex subject. But our note taking during last night's broadcast has left us woefully lacking and we wondered if you would be kind enough to tell us again the name of the ‘endings’ on the end of a chromosome. You illustrated this beautifully with a length of very thick rope which you produced not to scale because if you had done so it would have stretched as far as... Madrid!! How cool is that?!!’

Of course, it's easy to say that we're all too busy to get involved in public engagement, but this is important work.

All biologists have fantastic stories—so go tell them!
